# Estimated Cost of Anticancer Therapy Directed by Comprehensive Genomic Profiling in a Single-Center Study

**DOI:** 10.1200/PO.18.00074

**Published:** 2018-11-02

**Authors:** Anita Chawla, Filip Janku, Jennifer J. Wheler, Vincent A. Miller, Jason Ryan, Rachel Anhorn, Zhou Zhou, James Signorovitch

**Affiliations:** **Anita Chawla**, **Zhou Zhou**, and **James Signorovitch**, Analysis Group, Boston; **Vincent A. Miller**, **Jason Ryan**, and **Rachel Anhorn**, Foundation Medicine, Cambridge, MA; and **Filip Janku** and **Jennifer J. Wheler**, The University of Texas MD Anderson Cancer Center, Houston, TX.

## Abstract

**Purpose:**

Comprehensive genomic profiling (CGP) detects several classes of genomic alterations across numerous genes simultaneously and can match more patients with genomically targeted therapies than conventional molecular profiling. The current study estimated the costs of anticancer drugs and overall survival (OS) for patients who were treated with matched and unmatched therapy.

**Methods:**

Costs were estimated for patients with complete data (188 of 500 patients) from a prospective, nonrandomized study of patients with diverse refractory cancers who underwent CGP and were treated with matched or unmatched therapy. We assessed mean time to treatment failure (TTF) and mean observed OS. Patient-specific drug and administration costs were imputed for the first regimen after CGP on the basis of drug classes, unit costs, and time on treatment.

**Results:**

Patients on matched (n = 122) versus unmatched (n = 66) therapy had longer mean TTF (+1.5 months) and observed OS (+2.4 months) and higher drug costs (+$38,065; all *P* < .01). Increased drug costs were largely attributable to the longer duration of therapy associated with extended TTF (66.3%) rather than higher monthly drug costs (33.7%). Incremental increases in TTF (+1.9 months *v* +1.2 months) and observed OS (+2.5 months *v* +2.1 months) between matched and unmatched therapies were larger for those who underwent CGP in earlier- versus later-line therapy. Incremental increases in drug costs between matched and unmatched therapies were lower for earlier- compared with later-line therapy (+$27,000 *v* +$43,000, respectively).

**Conclusion:**

Matched therapy was associated with longer TTF, increased OS, and manageable incremental cost increases compared with unmatched therapy. Most of these increased costs were a result of the longer duration of therapy rather than higher monthly drug costs. The benefits of matching were numerically greater in earlier versus later lines of therapy, which is consistent with the value of early use of CGP.

## INTRODUCTION

Patients with metastatic cancer who have experienced failure with standard therapy often have few therapeutic options remaining; however, in recent years, the development of personalized medicine has shifted the oncology treatment landscape. This includes the use of next-generation sequencing (NGS) platforms that vastly improve DNA sequencing efficiency by allowing millions of reactions to occur in parallel.^1-4^ Many cancers have been shown to contain genomic alterations that can be targeted by specific therapies. Most recently, these targeted therapies have become a major area of focus in anticancer drug development.^5-15^ In 2017, for example, the US Food and Drug Administration (FDA) added five new targeted drugs and biologics to its list of more than 200 approved anticancer agents.^16,17^ Furthermore, recent National Comprehensive Cancer Network Clinical Practice Guidelines in Oncology for melanoma and non–small-cell lung cancer recommend broad molecular profiling to identify genomic alterations for matched therapy or to appropriately counsel patients with metastatic disease regarding the availability of clinical trials.^18,19^ In addition, the recently developed Precision Medicine Initiative includes the National Cancer Institute Molecular Analysis for Therapy Choice clinical trial, which seeks to determine the effectiveness of genomically targeted therapies.^20^ Furthermore, several prospective pantumor studies using comprehensive genomic profiling (CGP) have demonstrated improvements in clinical outcomes for patients who were treated with genomically matched therapy compared with those who received unmatched therapy.^7,12,13,21^ Recent evidence has also suggested that genomic testing at the time of diagnosis of metastatic disease, rather than after one or more lines of systemic therapy, may improve the chances for patients to receive clinically beneficial matched treatment before they undergo a rapid functional decline as they experience disease progression.^22^

The FDA issued its first approval of an NGS-based diagnostic test in December 2016, the FoundationFocus CDx_BRCA_ test (Foundation Medicine, Cambridge, MA), which matches patients with advanced ovarian cancer who have either germline (inherited) and/or somatic (acquired) *BRCA1/2* mutation types to treatment with the poly (ADP-ribose) polymerase inhibitor, rucaparib.^23^ In addition to testing for individual genomic alterations, use of CGP allows for the detection of four major classes of DNA alterations, including base substitutions, copy number alterations, insertions and deletions, and rearrangements. Collectively, the breadth and accuracy of CGP allow several potential targets for matched therapies to be identified in parallel regardless of anatomic site and without repeated site-specific tests.^3,4,24^ Therefore, CGP matches more patients with targeted therapies and may mitigate some of the key limitations of conventional molecular testing, including a lack of sufficient tissue because of the need for sequential testing, delays in receiving matched therapy, and an inability to detect actionable alterations.^25-30^ In 2017, FoundationOne CDx (Foundation Medicine), an NGS-based test that detects alterations in 324 genes, select rearrangements, and genomic signatures, such as microsatellite instability (MSI) and tumor mutational burden (TMB), became the first FDA-approved broad companion diagnostic for solid tumors. Although this diagnostic test is newly approved, there is a robust evidence base for the FoundationOne laboratory-developed test, and the evidence that supports the latter test can be considered transferrable to the FDA-approved FoundationOne CDx.

Although the clinical utility of genomically matched therapy has been established, questions remain about the value of CGP and the associated costs of targeted therapy. The costs of novel cancer drugs routinely exceed $100,000 per patient per year, with median costs ranging from $102,677 to $137,952, depending on the level of evidence available at approval.^31^ In patients with advanced solid tumors, costs associated with molecular-guided therapy totaled more than €31,000 per patient, with anticancer drugs and hospitalizations accounting for 54% and 35% of these costs, respectively.^32^ In a matched cohort study, patients who received targeted therapy experienced improved progression-free survival relative to the control group who received chemotherapy or best supportive care (22.9 weeks *v* 12 weeks; *P* < .05), whereas costs per weeks of progression-free survival did not differ significantly between groups ($4,665 *v* $5,000; *P* = .126).^33^ Furthermore, in a decision analytic model of patients with stage IV and recurrent incurable adenocarcinoma, first-line targeted therapy was demonstrated to be marginally cost effective with an incremental cost-effectiveness ratio of $110,658 per quality-adjusted life year compared with $122,234 per quality-adjusted life year in patients who underwent rebiopsy and chemotherapy.^34^ These findings suggest that, although targeted treatments may present high initial costs, such costs may be offset by the overall cost effectiveness of treatment and care throughout the course of disease.

In addition to the preliminary economic evidence currently available, additional studies are needed to explore the economic value of CGP in patients with advanced cancer. The objective of the current study was to estimate anticancer drug costs, time to treatment failure (TTF), and overall survival (OS) among patients with refractory cancers who had undergone CGP and were either matched or unmatched to targeted therapies. The analysis also further evaluates these outcomes by line of therapy.

## METHODS

### Patient Population

Our study is a post-hoc retrospective analysis of patients with diverse refractory tumors who were enrolled in a prospective, nonrandomized, phase I oncology center study. Detailed inclusion criteria have been described elsewhere.^10^ This study (ClinicalTrials.gov identifier: NCT02437617) was approved by the MD Anderson Cancer Center Internal Review Board, and all patients gave informed consent. Information collected by study investigators included age, sex, Eastern Cooperative Oncology Group performance status, tumor type, number of metastatic sites, and number of prior therapies. The FoundationOne 236-gene targeted sequencing panel was used to detect genomic alterations, including base substitutions, insertions and deletions, copy number alterations, and rearrangements. In addition, the specific drug classes used in the first regimen after CGP and the therapy type—matched or unmatched—were recorded. A drug was considered matched if the half-maximal inhibitory concentration impacted the target at low nanomolar range—for small-molecule inhibitors—or if the target was the primary one recognized by an antibody. Local therapy and transplantation were considered unevaluable for matching. The matching designation was confirmed by an investigator from the original phase 1 trial, who was blinded to patient outcome. Additional details on matching definitions are available in the primary clinical study manuscript.^10^

### Outcomes

We assessed clinical outcomes TTF and OS during the observation period as described previously.^10^ For this retrospective economic analysis, TTF and OS were calculated as observed cases—that is, not statistically adjusted. Mean monthly drug costs—adjusted to 2016 USD—were imputed for the first regimen used after CGP on the basis of unit costs of the individual drugs or representative agents of the drug classes and their label-based dosing schedules. Mean monthly drug administration costs (2016 USD) were imputed for intravenous drugs on the basis of Centers for Medicare & Medicaid Services fee schedule. In cases in which different formulations were available, the mean of the less costly formulation was used. Mean price per milligram was then multiplied by dose per day and days per month to calculate total cost per month. If multiple prices existed for a given drug with different indications, the cost associated with the closest cancer type was selected. If unclear, the mean value was used. Investigational drugs assumed no cost; administration costs were assumed to be $136.41 on the basis of Healthcare Common Procedure Coding System code 96413 for 1-hour infusion using Centers for Medicare & Medicaid Services physician fee schedule. Body surface area for infusions was assumed to be 1.79 m^2^, and body weight was assumed to be 80 kg. Total drug and administration costs were calculated as the product of mean monthly drug and administration costs and TTF.

### Statistical Analysis

The goal of this economic study was to calculate drug-related costs and their association with observed periods of treatment and survival to better understand cost drivers. Mean TTF, mean OS, and mean monthly and total drug and administration costs per patient were calculated over the observation period by therapy type—matched or unmatched. Differences between these groups were assessed using *t* tests.

To assess the drivers of drug cost differences between matched and unmatched therapies, the difference was partitioned into two components: the contribution of differences in treatment duration, calculated as the difference in mean durations for matched versus unmatched therapy multiplied by the monthly cost of unmatched therapy, and the contribution of differences in monthly treatment costs, calculated as the difference in monthly drug and administration costs for matched versus unmatched therapy multiplied by the mean duration of matched therapy. The contribution of each component was also calculated as a percentage of the overall difference in costs between matched and unmatched therapy. Analyses were conducted for the overall patient and population and also stratified by line of therapy of the first regimen after CGP—that is, earlier-line (one to three) versus later-line (four or later) therapy.

## RESULTS

### Patient Population

**Fig 1.**
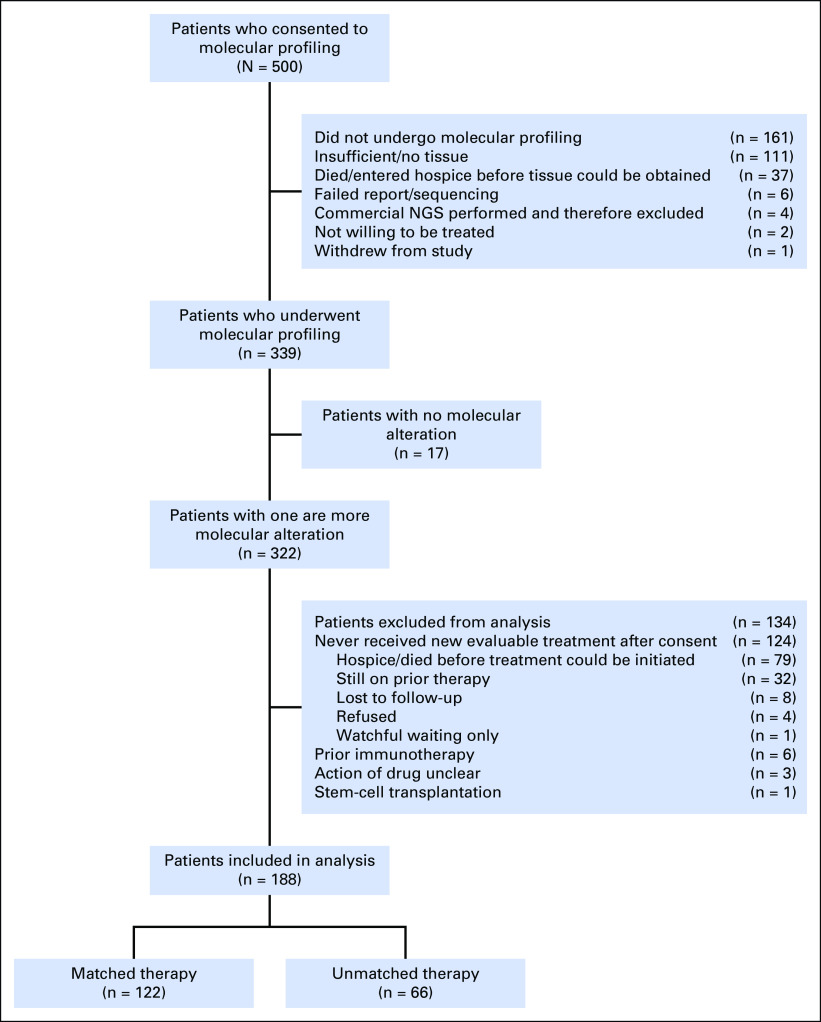
Patient flow diagram. NGS, next-generation sequencing. Adapted from Wheler et al^10^ with permission from the American Association for Cancer Research.

A total of 500 patients were enrolled. Among the initial enrollees, 161 did not undergo CGP, mostly for reasons of insufficient/no tissue, death, or referral to hospice before tissue could be obtained. Of the 339 patients who underwent molecular profiling, 322 had at least one genomic alteration detected—317 had one or more potentially actionable alteration—and 188 received subsequent treatment with either matched (n = 122; 65%) or unmatched (n = 66; 35%) therapy^10^ (Fig 1). Of the 134 patients with at least one alteration who were not included in the analysis, 124 never received a new evaluable treatment after providing consent, six were excluded as they had received prior immunotherapy, three received a drug with an unclear action, and one underwent stem-cell transplantation.^10^

Median age of treated patients was 59 years (range, 19 to 82 years), with 55% of patients age ≤ 60 years and 45% of patients older than age 60 years in both the matched and unmatched therapy groups. There were 42 (34%) and 24 (36%) men in the matched and unmatched therapy groups, respectively. Most clinical characteristics were well-balanced between groups, including Eastern Cooperative Oncology Group performance status and the number of prior lines of therapy. The number of molecular alterations identified per person was most frequently four (unmatched therapy) or five (matched therapy). Combination therapy was used as prior treatment(s) for 71% of matched and 53% of unmatched patients. Median TTF on prior lines was 2.6 months (range, 0.5 to 19.7 months) for matched and 3.0 months (range, 0.4 to 96.0 months) for unmatched patients (Table 1).

**Table 1.**
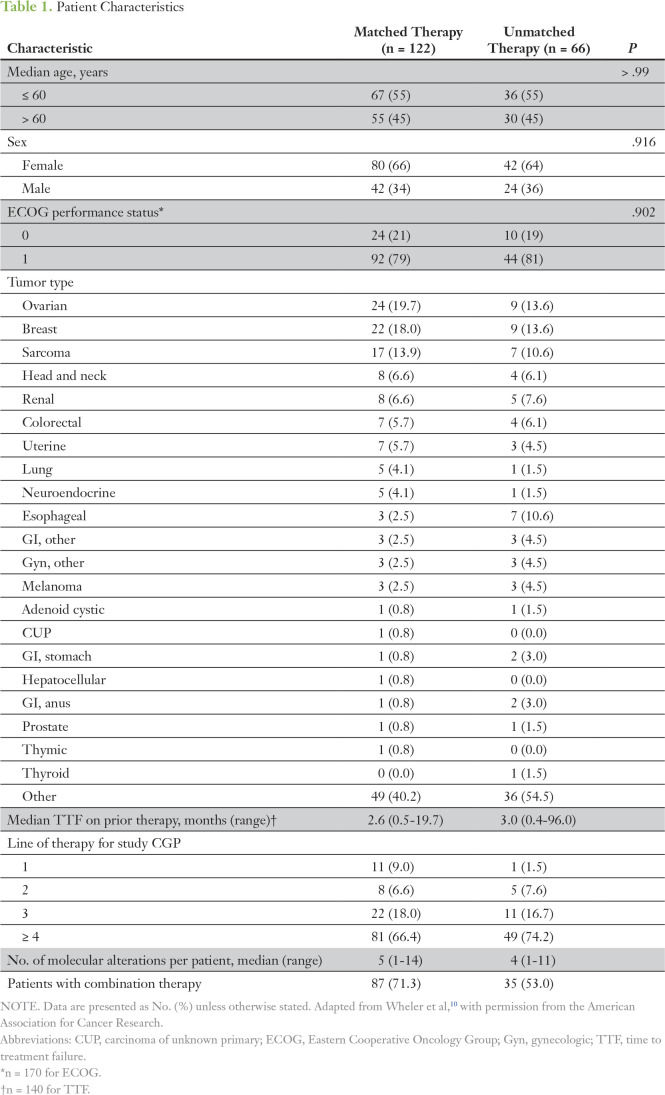
Patient Characteristics

### Patient Survival, TTF, and Drug Costs

#### Matched versus unmatched therapy.

**Fig 2.**
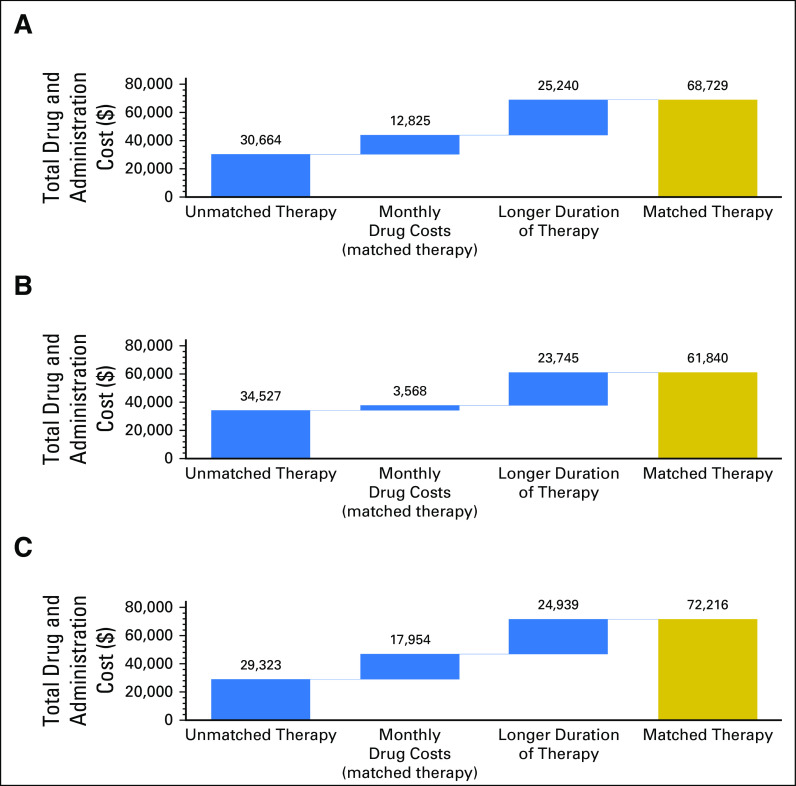
Comparison of total drug treatment costs between matched and unmatched therapy. (A) All lines. (B) Patients with one to three lines of prior therapy. (C) Patients with four or more lines of prior therapy.

In this retrospective analysis using observed cases, patients who received matched therapy demonstrated longer mean TTF (3.9 months *v* 2.4 months; *P =* .002), longer mean observed OS (8.2 months *v* 5.9 months; *P* < .002), and higher mean anticancer drug costs ($68,729 *v* $30,664; *P* = .003) compared with patients who received unmatched therapy (Table 2). Increased drug treatment costs for matched therapy were largely attributable to a longer duration of therapy (66.3% of costs), which was associated with longer TTF, rather than higher monthly drug costs (33.7% of costs; Fig 2). A total of 16 patients—nine who received matched therapy and seven who were unmatched—received an investigational agent that was assumed to have no cost; however, three of these patients received the agent in combination with either one or two costed drugs.

**Table 2.**
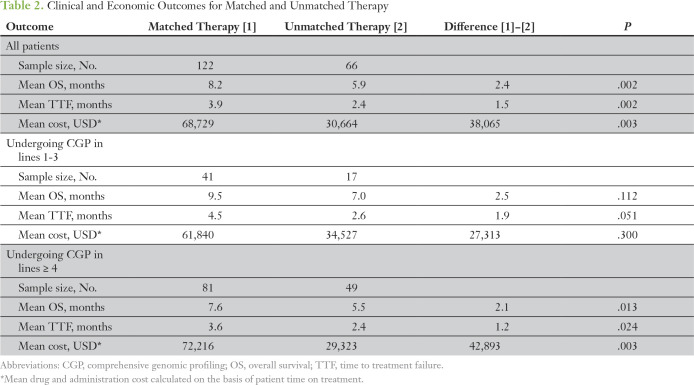
Clinical and Economic Outcomes for Matched and Unmatched Therapy

#### Earlier- versus later-line therapy.

The patient population who underwent CGP in earlier lines of therapy (lines one to three; n = 58) had a higher proportion of patients on matched therapy (70.7% *v* 62.3%) compared with the population who underwent CGP in later lines (lines four or later; n = 130). The distribution of tumor types under earlier- and later-line therapy is displayed in Appendix Table A1.

The incremental TTF between matched and unmatched therapies was numerically greater for those who underwent CGP in earlier lines of therapy compared with those who underwent CGP in later lines (1.9 *v* 1.2 incremental months). Likewise, the incremental OS between matched and unmatched therapies was also numerically greater for those who underwent CGP in earlier lines of therapy compared with those who underwent CGP in later lines (2.5 *v* 2.1 incremental months). In addition, the incremental increases in drug costs between matched and unmatched therapies were lower for earlier-compared with later-line therapy (approximately $27,000 *v* $43,000; Table 2). This was a result, in part, of the use of more costly regimens for later-line therapy, including more frequent use of combination therapies (77.8% in later-line therapy *v* 58.5% in earlier-line therapy).

Most of the incremental increases in drug treatment costs between matched and unmatched therapies were attributable to a longer time on treatment and survival in both earlier- and later-line therapies (87% and 58% of costs, respectively; Fig 2). In addition, the contributions of monthly drug costs to overall anticancer drug costs were much lower in earlier- versus later-line therapies (13% *v* 42%, respectively).

## DISCUSSION

To our knowledge, this is the first study to report anticancer drug costs for matched and unmatched therapies in patients with refractory cancer who have undergone CGP. Patients who were treated with matched therapy generally had a longer time on treatment, greater observed OS, and higher anticancer drug costs compared with those on unmatched therapy. It is also notable that patients in the matched group experienced these improved outcomes despite having an approximately 6-month lower median TTF on their prior therapy compared with the unmatched group, which suggests that their disease had been comparatively less responsive to the last line of treatment these patients had received. Most of these increased drug treatment costs were attributable to a longer time on treatment and improved survival rather than higher monthly drug costs. The benefits of matched therapy may therefore outweigh the increased costs.

In addition, our analysis found more promising outcomes for matched therapies when administered to a patient population undergoing CGP in earlier lines of therapy. A higher proportion of patients who underwent CGP in earlier lines was administered matched therapies compared with those who underwent CGP in later lines, a finding consistent with previously published literature.^22^ Those who underwent CGP in earlier-line therapy also had incremental increases in TTF and OS that were numerically larger for matched versus unmatched therapy compared with patients who underwent CGP in later-line therapy.

Of importance, patients tested in earlier-line therapies had a lower increase in drug costs from unmatched to matched therapy compared with patients who received testing in later-line therapy ($27,000 *v* $43,000). Similar to the analysis conducted on the full sample, most of the incremental increases in drug treatment costs were attributable to longer TTF and survival in both earlier- and later-line therapies. Because incremental costs were numerically lower per patient in the earlier- versus later-line group, despite their increased OS and TTF, these findings suggest that earlier use of CGP could enable matching to effective treatment options that have lower costs than those used in later lines.

Higher anticancer drug costs for matched therapy have been reported elsewhere.^32^ In this study, however, the observed high costs may have been a result of patients undergoing CGP in later- as opposed to earlier-line therapies, as more than 93% of the patient population had previously received chemotherapy or a targeted therapy. Furthermore, the study authors did not detail the individual factors that underpinned anticancer costs, such as a longer time on treatment, which represented 66.3% of the cost increase in our study, rather than higher monthly drug costs.

### Limitations

Although this retrospective study estimates the anticancer drug costs associated with matched and unmatched therapies, its contribution must be balanced with the limitations of the data. For example, most patients were later line, and therefore the effects of CGP in lines one and two were underrepresented. In addition, three tumor types—ovarian, breast, and sarcoma—represented more than 50% of tumors. It is also important to note that care at a phase I clinic at a premier care center may not represent the level of drug treatment accessible to patients in community practice, especially those who receive later lines of therapy. These findings can thus be viewed as representing the upper range of the economic impact of CGP. Another notable limitation is that the study does not account for the value of CGP in informing the use of checkpoint inhibitors because it was conducted before their approval. Of note, TMB, which can be assessed using FoundationOne and FoundationOne CDx, has recently been shown to be predictive of outcomes among patients who are administered checkpoint inhibitors.^35^ Indeed, both TMB and MSI were not well-established as markers for targeted therapy during the time period of the phase I study,^10^ whereas pembrolizumab is now approved for patients with unresectable or metastatic solid tumors with MSI high or mismatch repair deficiency. Overall, the rapidly evolving precision medicine landscape in oncology underpins the strong potential for increased clinical utility as more patients have the opportunity to become matched to targeted therapy.

In addition, this analysis included phase I drugs that may not have engaged the target or had poor pharmacokinetics, and patients who received all dose levels were also included, which may reduce the positive effect of treatment.^10^ Furthermore, drug acquisition and administration costs were estimated on the basis of list prices and durations of therapy, and costs for medical services were not included. Analyses that were based on actual costs would be preferable if such data were available. Our analysis was also limited by the relatively high number of patients who could not be administered therapy during the study as a result of the unpredictable and rapid disease course of refractory cancers. Among included patients, it was not possible to separate out the possible impacts of line of therapy and performance status on the differential OS and TTF findings.^10^ Finally, patients were assumed to take the dose of each drug according to the prescribing information, which would not account for potential dose reduction in combination therapies.

In conclusion, CGP is useful for selecting patients with refractory tumors to receive matched therapy. This study demonstrates longer treatment durations, longer survival times, and manageable incremental increases in costs among patients undergoing matched therapy compared with unmatched therapy. Furthermore, most of the increased costs of matched therapy were a result of longer treatment times rather than higher monthly drug costs. Findings also suggest that there could be a greater value associated with early-line use of CGP to guide treatment among patients with refractory tumors. Collectively, these results demonstrate the considerable opportunity that exists for CGP to improve treatment strategies in patients with refractory cancers.

## Figures and Tables

**Table A1.**
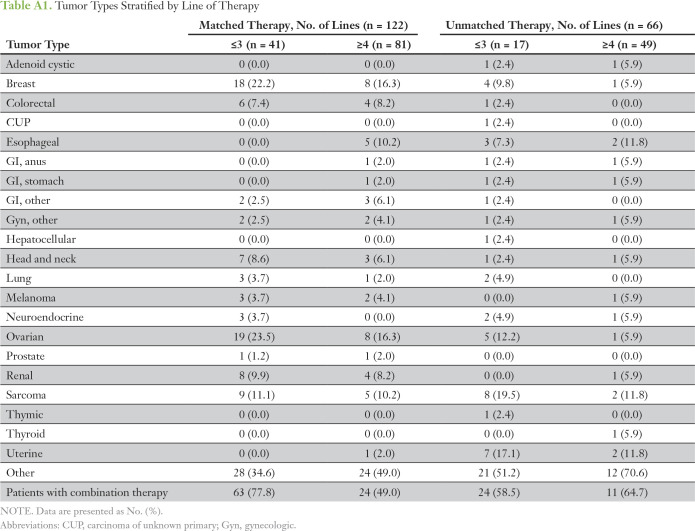
Tumor Types Stratified by Line of Therapy
